# Retention Protocols and Factors Affecting Retainer Choice among Iraqi Orthodontists

**DOI:** 10.1155/2020/8810641

**Published:** 2020-10-23

**Authors:** Mushriq F. Abid, Ali M. Al-Attar, Akram F. Alhuwaizi

**Affiliations:** Orthodontic Department, College of Dentistry-Baghdad University-Iraq, Baghdad, Iraq

## Abstract

**Background:**

To identify the most common retention protocols practiced by Iraqi orthodontists using a specially designed e-survey. Furthermore, this study aimed to assess the effect of sociodemographic factors on the participant's choice.

**Methods:**

Two hundred and twenty-five questionnaires with 23 multiple choice questions were sent to members of the Iraqi Orthodontic Society. The questionnaire was organized into four sections representing information about sociodemographic status of the orthodontists, factors affecting the selection of the retention system, commonly used retainers in the upper arch and lower arch, and duration of the retention system. The chi-square test was used to test the significant association between different variable and sociodemographic factors.

**Results:**

The response rate was 87.5%. The majority of the respondents considered the original malocclusion (80.2%) and clinical experience (49.7%) as the main factors for choosing the retention protocol. In the maxillary arch, a combination of vacuum-formed retainer and fixed retainer (35%) was mostly applied; in the mandibular arch, a fixed retainer was mainly used (46.7%). Most of the respondents recommended initial full-time wearing of a removable appliance (78.2%), especially in the first 3–6 months (47.2%). According to the respondents, bonding a fixed retainer to all anterior teeth was most common (79.7%), fabricated, and adapted directly inside the patient's mouth (75.1%). More than half used flowable composite (54.8%) and recommend leaving the retainer forever (53.8%). Most of the variables showed a statistically significant association between the sociodemographic factors and type, duration, and fabrication of the retainer used.

**Conclusions:**

A combination of removable and fixed retainers was commonly used in orthodontics retention, and sociodemographic factors significantly affected retainer choice.

## 1. Introduction

Prevention of relapse and maintenance of the orthodontic treatment require the use of retention protocols and appliances [1]. Relapse can occur at any age, and it is most commonly caused by occlusal forces, soft tissue pressure, and continuous growth, which could be influencing factors [2]. Surprisingly, to date, there has been no universal agreement about which retention protocol should be recommended including the need for retention, the type of retainer needed, and retention duration. However, several systematic and Cochrane reviews concluded that there is insufficient evidence to make recommendations on orthodontic retention regimen [3, 4].

Many types of orthodontic retainers are used after orthodontic treatment including removable acrylic retainers, vacuum-formed retainers (VFRs), and bonded retainers. It seems that the choice for a orthodontic retainer type is still debatable due to a large number of variations in retention materials, strategies, and individual patient factors. Orthodontists in the US mainly prefer the use of Hawley or VFR in the maxillary arch and the bonded retainer in the lower arch [5]. In Norway, they use a combination of bonded and removable retainers in the upper arch, while bonded retainers are used in the lower arch [6]. In Ireland, orthodontists recommend full-time wear of VFR in the maxillary and mandibular arches followed by part-time wear [7]. There are several disadvantages of different types of retainers, for example, a bonded retainer could cause plaque accumulation, poor oral hygiene, and influence the periodontal health. Detrimental periodontal health could have a serious consequence on body health, since an association periodontitis and coronary heart disease have been reported [8]. Nevertheless, a recent study reported that using nutraceutical agents is effective in reducing periodontal parameters and controlling the level of pain and inflammatory mediators in patients with periodontitis [9].

To date, several surveys of the retention protocols and trends used in different countries have been conducted revealing preferences and differences between the orthodontists [10–16]. Furthermore, studies are required for the development of retention protocol, which would be useful in finding a retention regime that can be used by orthodontists around the world. For this reason, this study aimed to evaluate retention trends and protocols used by Iraqi orthodontists, most commonly used types of retainers in the upper and lower dental arches, and identify the effect of sociodemographic factors on retainer choice.

The null hypothesis: we expect that no specific type of retainer is commonly used in the upper or lower dental arches; moreover, sociodemographic factors have no effect on retainer choice.

## 2. Materials and Methods

This was a cross-sectional study in the form of an e-survey involving orthodontists who are active members of the Iraqi Orthodontic Society. The survey took ten weeks from February 2020 till April 2020, and orthodontists were invited to fill out an online questionnaire (Google Form) of 23 questions. The participants were advised to contact the authors for inquiries related to answering the questionnaire. The procedure and protocol of the present study were approved by College of Dentistry, University of Baghdad, in accordance with the Helsinki Declaration for human research studies (reference no. 192420).

The sample size was calculated according to the following formula:

(1) $$\begin{array}{c} N=\frac{\left(N/1\right)+Z2\;x\;P\left(1-P\right)}{E2N}, \end{array}$$

where *N* is the population size, *Z* is the *z* score for % confidence interval, *E* is the margin of error, and *P* is the population proportion (0.5)

Thus, the calculated sample size was equal to 152 at 95 confidence interval and 5% margin of error. To avoid potential dropout, additional 15% (23 subjects) was added to the sample; then, the final sample size was rounded to 175 subjects.

The questionnaire was adapted from two previous studies [15, 17], and it was adjusted to include additional aspects. The preliminary survey was pilot tested on 15 academic and experienced orthodontists; consequently, the questions were reviewed and modified to the final form of the questionnaire. The questionnaires were sent to the members via emails at least three times to maximize the response rate. The questionnaire (Supplementary Materials (Supplementary Materials (available here)) was divided into four parts representing some specific information as follows:

First, the participants were asked about their gender, the duration of clinical experience, and the location of clinical practice (work sector). The second part of the questionnaire included questions about the types of retainers used in the maxillary and mandibular arches, factors influencing the selection of the retainer type, factors influencing the choice of retention protocol, cases the participants find mostly susceptible to relapse and need longer retention period, adjunctive treatment frequently used to increase stability, and the type of retainer mostly preferred by the patient.

The third section included information on the number of checkups after placing a removable retainer, initial wearing time, the total duration of wearing the retainer, recommendation of the participants for the full-time wear of the appliance, and when do the participants recommend stopping the retention. The last section referred to the most common indication of the fixed retainer, bonding type of the fixed retainer, contraindication of the fixed retainer, method of fabrication of the fixed retainer, time of removal of the fixed retainer, type of wire used, number of checkups after fixed appliance placement, how often the participants observe failure of the fixed retainer, and type of bonding material used to fix the retainer.

Both descriptive and inferential statistics were performed for analyzing the data. Descriptive statistics were performed to define all categorical data in the form of counts and percentages. Chi-square was used to test the significant relationship between sociodemographic factors and all other variables as follows: (questions 1–3) and questions related to selection of the retention system (questions 4–10) and removable retainers (questions 11–14) and fixed retainers (questions 15–23). *P* values ≤ 0.05 were considered statistically significant.

## 3. Results

Two hundred and twenty-five questionnaires were sent out, and one hundred and ninety-seven were returned completed giving a response rate of 87.5%. More than half of the respondents were males (*n* = 115, 58%). The majority of the respondents worked in the private practice sector (*n* = 151, 76.6%), while (*n* = 17, 8.6%) some of the respondents worked at the university. Regarding work experience, about half of the respondents had more than ten years of experience (*n* = 92, 46%) and about quarter of them had less than five years' experience (*n* = 50, 25.4%) (Table 1).

In the second part of the survey, the majority of respondents answered that the original malocclusion is the main factor for selecting the retention system (*n* = 158, 80.2%). More than half of the respondents (*n* = 116, 58.9%) selected teeth spacing as the case needing longer retention. About half of the orthodontists (*n* = 98, 49.7%) chose clinical experience as an influencing factor for selecting the retention system. More than half of the respondents (*n* = 104, 52.8%) used overcorrection as an adjunctive method for increasing stability. Fixed retainers were preferred by most of the patients (*n* = 84, 42.6%), while only (*n* = 1, 2%) few preferred the Hawley retainer. The most common type of retainer used in the upper arch was a combination of the VFR and fixed retainer (*n* = 69, 35%), followed by VFR (*n* = 64, 32.5%). However, about half of the orthodontists used a fixed retainer in the lower arch (*n* = 92, 46.7%) followed by vacuum-formed and fixed retainers (*n* = 53, 26.9%) (Table 2).

Regarding the third part of the survey, the majority of the orthodontists recommended initial full-time wearing of the appliance (*n* = 154, 78.2%). Almost half of the respondents recommended 3–6 months' full-time wear (*n* = 93, 47.2%). More than one-third of the orthodontists check the patients more than three times throughout the retention period (*n* = 75, 38.1%). Moreover, more than half of the orthodontists (*n* = 106, 53.8%) stop the retention less than two years after debonding and 30.5% more than two years, while 10.2% recommended long-term wear of the appliance (Table 3).

Finally, in the last section of the survey, the majority of the respondents recommended fixed retainers in patients with spacing (*n* = 151, 76.6%). Additionally, most of them preferred a fixed retainer bonded to all anterior teeth (*n* = 157, 79.7%). The most frequently mentioned contraindication for fixed retainers were poor oral hygiene, periodontal problem, and caries (*n* = 129, 65.5%). The majority of the orthodontists fabricate the retainer directly inside the patient mouth (*n* = 148, 75.1%). The most common type of wire used is the dead soft multistrand wire (*n* = 66, 33.5%), and more than half of the orthodontists used flowable composite for fixing the retainer (*n* = 108, 54.8%). More than half of the orthodontists check the retainer on breakage or debonding of the fixed retainer (*n* = 103, 52.3%). More than one-third of the orthodontists observed failure of the fixed retainer twice during the retention period (*n* = 67, 34%). More than half of the orthodontists recommended wearing the retainer forever (*n* = 106, 53.8%) (Table 4).

Table 5 illustrates chi-square test results. Interestingly, most of the variables showed a statistically significant association between the sociodemographic factors and the variables selected. In the upper arch, males chose VFR, while females selected a combination of VFR and fixed retainers, which was also selected by orthodontists who work at universities and have an experience of more than 10 years. While, in the lower arch, males selected fixed retainer, and females orthodontists who work at the universities and experienced orthodontists selected a combination of VFR and fixed retainers.

Regarding factors affecting retainer choice, males and orthodontists with more than 10 years' experience chose clinical experience, while females and orthodontists who work at MOH chose knowledge from books. Most males, those who worked at private clinics and those with 5–10 years' experience, stopped retention with a removable appliance in less than two years. With a fixed retainer, most males and private sector orthodontists bond the retainer from canine to canine, and most females, those in the university work sector, and more experienced orthodontists recommended wearing the retainer forever.

## 4. Discussion

To date, there have been different types of fixed and removable retainers; however, it is still unclear which type is the best and how long they should be kept [3]. Previous studies have reported that the retention regimen is affected by different factors such as severity and type of malocclusion, patient age, and compliance [15, 18]. Common retention trends among orthodontists practicing in different countries are probably influenced by experience, training background, oral health attitude, and dental care delivery [5, 6, 13, 14]. This study was planned to investigate the retention protocols and the effect of demographic factors on retention choice among orthodontists.

The response rate in the present study was relatively high (87.5%) when compared to previous surveys conducted in other countries [7, 10–14, 19]. Although orthodontists in the present study chose different types of retainers and different orthodontic malocclusion, some trends were observed. A combination of a vacuum-formed retainer and fixed retainer was most commonly used in the upper arch followed by VFR (35% and 32.5%, respectively), which was reported by a previous Norway study [6]. This outcome is contrary to previous studies; orthodontists in the Netherlands and Switzerland mostly used fixed retainers [12, 13], while Hawley retainers were commonly used in the USA and Saudi Arabia [5, 14]. VFRs were most often chosen by UK, Ireland, and Malaysian orthodontists [7, 11, 19]. Atack et al. reported that relapse in the lower anterior teeth can occur with both removable and fixed retainers, which could justify the use of a double retention system to prevent relapse [20]. Interestingly, fixed and VFRs were preferred by the patients (42.6% and 36%, respectively), which could be attributed to the fixed retainer requiring minimal patient compliance. In the lower arch, most of the respondents selected a fixed retainer, which is in accordance with most previous studies [11–14]. Conversely, VFRs were dominant in other countries [7, 19]. It is worthy of mention that recently, it has been reported that clear aligners can be used for retention, and one of their advantages is a global improvement of facial aesthetics [21].

Most of the orthodontists in the present study selected malocclusion, particularly spacing and clinical experience as influencing factors for selecting the retention system. Similar findings were reported from previous surveys [6, 8]; previous research studies on the relationship between malocclusion and posttreatment stability and relapse are controversial. Some studies fail to find a significant correlation [22, 23], while other studies reported more relapse in severe malocclusion [24]. Recent reports supported this controversy and reported no significant association between relapse with either the type of retainer or original malocclusion [25, 26]. More than half of the respondents chose overcorrection as an adjunctive method to enhance retention, and 19.3% preferred interproximal stripping (IPR). However, no adjunctive procedure was reported by 12.7% of the orthodontists who tend to recommend lifetime retention. Al-Jewair et al. reported that IPR was more common among Saudi orthodontists and with 26.8%, no adjunctive procedure was performed [14].

In the present study, most of the orthodontists recommended initial full-time wearing of the appliances for at least 3–6 months, which accords with most of the previous studies [14, 17]. This is regardless of the type of retainer whether it is vacuum-formed or Hawley retainer, on the contrary, Sheridan et al. suggested night wear of vacuum-formed retainers to prevent open bite [27]. Moreover, other studies reported that gingival and periodontal fibers in rotated teeth are partially rearranged after eight months [28], which might explain the differences in the orthodontists choice on the duration of wear. More than half of the respondents recommended stopping the retention in less than two years and 30.5% recommended more than two years. Similarly, Lithuanian orthodontists recommended one to two years or five years and more [15], while other studies recommended lifetime retention [17] and a retention period of more than two years [10]. Long-term retention and frequent checkups are recommended for orthodontic patients to prevent relapse especially in the lower anterior teeth and changes in occlusion [29].

About two-thirds of the orthodontists preferred to bond the retainer to all anterior teeth, and the results of the present study were in line with most previous studies [15, 30]. Bonding a retainer to all anterior teeth was considered to be effective and ensure alignment of anterior teeth [31]. Nevertheless, these types of retainers could cause plaque accumulation, poor oral hygiene, and influence the periodontal health [32]. These disadvantages would justify why most of the respondents contraindicated fixed retainers in patients with poor oral hygiene and caries. The majority of orthodontists recommended lifetime wearing of a fixed retainer, which accords with most previous studies [10, 16, 30]. Littlewood suggested long-term retention to reduce posttreatment relapse [33]. The majority of the orthodontists fabricates and adapts the retainer inside the patient's mouth using the multistrand dead soft wire and flowable composite. Similarly, most Dutch orthodontists selected the multistrand dead soft wire [16], while Arnold et al. reported that single and multistrand stainless steel wires were most commonly used [34]. Moreover, it has been reported that flowable composite can be used as an alternative when compared to resin composite [35]. In contrast to other studies, which reported less shear bond strength of flowable composite [36], this could explain why one-third of the orthodontists observed failure of the fixed retainer twice during the retention period.

In the present study, chi-square showed a statistically significant difference among sociodemographic factors and retention protocol. For example, in the upper arch, a combination of a vacuum-formed retainer and fixed retainer was used by females more than males. This accords with other reports [6] and contradicts others who found that more male orthodontists are using fixed retainers [17]. It is difficult to explain why there are gender differences in choosing the retention protocol; however, some studies reported that female orthodontists spend fewer hours in practice and work fewer days, which could justify the different choices they made [37]. Interestingly, the place of work affected the orthodontists' choices of most of the responses. It appeared that orthodontists who work in the academic field follow certain protocols when compared to those who work in private clinics who were more daring in using current trends. Years of experience is an additional factor in affecting the retention protocol; recently, qualified orthodontists used more contemporary retention protocols when compared to senior clinicians who used traditional techniques, which is in accordance with a previous study [38].

### 4.1. Weakness and Strength of the Study

In the present study, the sample size and response rate were strong points, which could indicate that the study outcomes were representatives of the orthodontists in Iraq. However, the questionnaire did not involve all the questions about the details in retention protocols, but it was expected that more questions would have affected the response rate.

## 5. Conclusions

The most commonly used retainers among Iraqi orthodontists in the upper arch is a combination of vacuum-formed retainers and fixed retainers, while a fixed retainer was predominant in the lower arch. Original malocclusion and clinical experience were effective in selecting the retention protocol. Sociodemographic factors significantly affected the choice of retention protocol. The results of the present study may allow clinicians to compare their practice to others with similar gender, work sector, and experience.

## Figures and Tables

**Table 1:** Sociodemographic data.

Variables	No.	S%
Q1) Gender		
Male	115	58.4
Female	82	41.6

Q2) Practice duration		
<5 years	50	25.4
5–10 years	55	27.9
>10 years	92	46.7

Q3) Where do you mostly practice clinically?		
Private clinic	151	76.6
Ministry of health (MOH)	29	14.7
College clinic	17	8.60

**Table 2:** Selection of retention protocol (%).

	Gender^*∗*^		Work sector^*∗*^^*∗*^			Practice (years)				Total
	M	F	Private	MOH	College	<5		5–10	>10	
Q4) Selection of a retention system mostly depends on										
Original malocclusion	88	70	88	55	53	84		82	77	80
Oral hygiene and patient motivation	10	20	7	41	29	10		13	17	14
Patient age	2	9	4	3	12	4		5	4	5
Request of parents/patient	0	2	1	0	6	2		0	1	1

Q5) Case you find most susceptible to relapse and need a longer retention period										
Cl II div 1	10	1	7	3	0		14	4	3	6
Cl II div 2	3	1	3	0	0		6	0	1	2
Cl III	3	0	2	3	0		2	0	3	2
Teeth spacing	58	60	63	52	35		38	76	60	59
Teeth rotation	17	7	12	14	18		28	9	7	13
Open bite	9	30	13	28	47		10	11	26	18
Deep bite	1	0	1	0	0		2	0	0	1

Q6) Type of retainer commonly used in the upper arch										
Hawley	3	4	3	10	0		8	2	2	4
VFR^*∗*^^*∗*^^*∗*^	41	21	36	24	18		46	36	23	32
Fixed retainer	26	6	21	3	18		12	29	14	18
Hawley and fixed retainers	13	9	13	7	0		6	9	15	11
VFR^*∗*^^*∗*^^*∗*^ and fixed retainers	17	61	28	55	65		28	24	46	35

Q7) Type of retainer commonly used in the lower arch										
Hawley	2	1	1	3	0		2	4	0	2
VFR^*∗*^^*∗*^^*∗*^	25	10	20	17	12		40	9	13	19
Fixed retainer	59	29	54	21	24		36	60	45	47
Hawley and fixed retainers	1	13	6	10	0		6	7	5	6
VFR^*∗*^^*∗*^^*∗*^ and fixed retainers	13	46	19	48	65		16	20	37	27

Q8) Factors influencing the choice of a retention protocol										
Knowledge gained from orthodontic books	16	46	20	62	47		20	24	36	28
Clinical experience	57	39	57	28	24		28	62	54	50
Knowledge and skills gained in postgraduate studies	21	13	20	3	24		44	11	8	18
Knowledge gained from the Internet	6	1	3	7	6		8	4	2	4

Q9) Adjudicative treatment you frequently use to increase stability										
Over correction	48	60	52	66	35		44	64	51	53
Interproximal stripping	25	11	23	3	12		40	16	10	19
Circumferential incision	17	12	17	10	6		12	16	16	15
None	10	17	7	21	47		4	4	23	13

Q10) Type of retainer mostly preferred by your patients										
VFR^*∗*^^*∗*^^*∗*^ retainer	40	30	40	31	12		52	42	24	36
Fixed retainer	49	34	45	38	29		16	51	52	43
Hawley retainer	2	0	1	0	0		4	0	0	1
Combination of VF^*∗*^^*∗*^^*∗*^ and fixed retainers	7	30	10	28	59		24	7	18	17
Combination of Hawley and fixed retainers	3	5	4	3	0		4	0	5	4

^*∗*^ *M*, male; *F*, females; ^*∗*^^*∗*^MOH, Ministry of Health; ^*∗*^^*∗*^^*∗*^VFR, vacuum-formed retainer.

**Table 3:** Removable retainers (%).

	Gender^*∗*^		Work sector^*∗*^^*∗*^			Practice (years)			Total
	M	F	Private	MOH	College	<5	5–10	>10	
Q11) Number of checkups after placing a removable retainer									
1	10	1	7	7	6	12	7	3	7
2	13	7	12	3	12	20	5	9	11
3	21	12	19	10	12	32	18	9	17
More than 3	42	33	42	31	12	20	45	43	38
On breakage or damage of the retainer	14	46	20	48	59	16	24	36	27

Q12) Initial wearing times of the removable retainer									
Full-time	83	71	85	59	47	90	89	65	78
After school, evening, and night	9	21	8	41	18	4	7	23	14
Evening and night	4	6	4	0	24	6	2	7	5
Nighttime only	3	2	3	0	12	0	2	5	3

Q13) How long do you usually recommend the removable retainer full-time wear?									
<3 months	13	5	12	0	6	16	7	8	10
3–6months	57	33	52	34	29	40	67	39	47
7 months-one year	22	59	30	59	65	34	22	48	37
>1 year	8	4	7	7	0	10	4	5	6

Q14) Usually when do you stop retention with removable retainers?									
<2 years after debonding	63	40	61	28	35	68	73	35	54
>2 years after debonding	27	35	29	45	18	22	15	45	30
After 3rd molars have erupted or extracted	1	12	3	21	6	2	2	10	6
Wear retainers forever	9	12	7	7	41	8	11	11	10

^*∗*^ *M*, male; *F*, females; ^*∗*^^*∗*^MOH = Ministry of Health.

**Table 4:** Fixed retainer (%).

	Gender^*∗*^		Work sector^*∗*^^*∗*^			Practice (years)				Total
	M	F	Private	MOH	College	<5	5–10	>10		
Q15) Most common indication of a fixed retainer is after treatment of									
Spacing	80	72	85	62	29	80	87	68		77
Crowding	9	6	9	3	0	14	4	7		8
Rotation	9	18	4	34	53	6	9	18		13
Open bite	3	4	2	0	18	0	0	7		8

Q16) The most preferred fixed retainer used is									
Bonded to four incisors	5	7	7	3	6	12	4	4		6
Bonded to canines and incisors	86	71	84	76	47	80	78	80		80
Bonded from first premolar to first premolar	9	17	8	14	47	6	16	13		12
Bonded to canines only	0	5	1	7	0	2	2	2		2

Q17) The most frequent contraindications for fixed retainers									
Poor oral hygiene, periodontal problems, and caries	77	50	72	52	35	78	71	55		65
Occlusion (deep bite)	19	33	21	48	18	18	18	33		25
Time, cost, and maintenance of fixation	3	17	7	0	47	2	11	12		9
Patient motivation	1	0	1	0	0	2	0	0		1

Q18) Method of fabrication of the fixed retainer									
Directly inside the patient mouth	82	66	84	52	35	76	85		68	75
On study cast by orthodontist	17	34	15	48	65	22	15		30	24
On study cast by technician	2	0	1	0	0	2	0		1	1

Q19) Usually when do you remove fixed retainers?									
Wear retainers forever	41	72	49	69	71	46	51		60	54
>2 years after debonding	29	17	26	17	12	22	27		23	24
<2 years after debonding	19	6	15	7	18	30	7		9	14
After 3rd molars have erupted or extracted	11	5	10	7	0	2	15		9	9

Q20) Most common wire used as a fixed retainer is									
Dead soft multistrand wire	25	45	28	45	59	22	20		48	34
Multistrand rectangular SS wire	31	29	33	24	18	48	38		16	30
Multistrand round SS wire	37	23	32	31	24	18	40		34	31
Chain	5	1	5	0	0	8	2		2	4
Glass fiber	1	1	1	0	0	4	0		0	1

Q21) Number of checkups after placement of the fixed retainer									
Once	3	0	1	3	6	4	0		2	2
Twice	8	4	5	7	12	10	5		4	6
Three times	22	11	19	7	18	30	16		11	17
More than three	29	13	26	14	0	20	31		18	22
On breakage or debond of the retainer	38	72	48	69	65	36	47		64	52

Q22) How often do you usually observe failure of a fixed retainer?									
Twice during the whole retention period	37	23	34	21	18	44	36		21	31
Once during the whole retention period	38	28	38	17	24	18	42		38	34
More than twice during the whole retention period	13	37	15	48	53	10	15		35	23
Never	12	12	13	14	6	28	7		7	12

Q23) Type of bonding material used to fix the fixed retainer									
Flowable composite	62	45	60	34	47	78	67		35	55
Orthodontic bonding material	13	39	18	45	41	10	5		42	24
Normal composite	25	16	23	21	12	12	27		23	21

^*∗*^ *M*, male; *F*, female; ^*∗*^^*∗*^MOH, Ministry of Health.

**Table 5:** Table of the chi-square test.

Question	Gender	Work sector	Years
Q4) Selection of a retention system mostly depends on	0.008	0.000	0.454
Q5) Cases most susceptible to relapse and need a longer retention period	0.000	0.004	0.000
Q6) Type of retainer commonly used in the upper arch	0.000	0.001	0.003
Q7) Type of retainer commonly used in the lower arch	0.000	0.000	0.000
Q8) Factors influencing the choice of a retention protocol	0.000	0.000	0.000
Q9) Adjudicative treatment you frequently use to increase stability	0.026	0.000	0.000
Q10) Type of the retainer mostly preferred by your patients	0.000	0.000	0.000
Q11) Number of checkups after placing a removable retainer	0.000	0.000	0.000
Q!2) Initial wearing times of the removable retainer	0.048	0.000	0.000
Q13) How long do you usually recommend a removable retainer full-time wear?	0.000	0.002	0.004
Q14) Usually when do you stop retention with removable retainers?	0.001	0.000	0.000
Q15) Most common indication of a fixed retainer is after treatment of	0.116^*∗*^	0.000	0.027
Q16) The most preferred fixed retainer used is	0.108	0.003	0.255^*∗*^
Q 17) The most frequent contraindications for fixed retainers	0.000	0.003	0.029
Q18) Method of fabrication of the fixed retainer	0.009	0.000	0.081^*∗*^
Q19) Usually when do you remove the fixed retainers?	0.000	0.099^*∗*^	0.003
Q20) Most common wire used as a fixed retainer is	0.013	0.128^*∗*^	0.000
Q21) Number of checkups after placement of a fixed retainer	0.000	0.013	0.007
Q22) How often do you usually observe failure of a fixed retainer?	0.001	0.000	0.000
Q23) Type of bonding material used to fix the fixed retainer	0.000	0.002	0.000

^*∗*^Not significant.

## Data Availability

The data used to support this study are made available from the corresponding author upon request.

## Supplementary Materials

Supplementary Materials The questionnaire form used in the study.
