# Supplementary material for: Retention Protocols and Factors Affecting Retainer Choice among Iraqi Orthodontists
Source: Int J Dent. 2020 Oct 23;2020:8810641. doi: 10.1155/2020/8810641 (PMC7603596; doi:10.1155/2020/8810641)
Supplement: Supplementary Materials — The questionnaire form used in the study. [file 8810641.f1.docx]

**Appendix (Questionnaire form)**

**All information of this survey will be used for academic purpose. Your responses will be anonymous and all personal information will be dealt with confidentiality. If yoy are willing to participate in this survey, please tick the box**

1. **Gender**

- Male.
- Female.

1. **Where do you mostly practice clinically?**

- Private clinic.
- Ministry of Health (MOH).
- College clinics.
- Other

1. **Duration of the profession practice**

- <5 years
- 5-10 years
- >10 years

1. **Selection of a retention system mostly depends on:**

- Original malocclusion
- Oral hygiene and patient motivation
- Patient age
- Request of parents/patient
- Other --------

1. **Case you find most susceptible to relapse and need longer retention period:**

- Cl II div 1
- Cl II div 2
- Cl III
- Teeth spacing
- Teeth rotation
- Open bite
- Deep bite
- Other -------

1. **Type of retainer commonly used in upper arch:**

- Hawley
- Essix
- Fixed
- Hawley and fixed
- Essix and fixed

1. **Type of retainer commonly used in lower arch:**

- Hawley
- Essix
- Fixed
- Hawley and fixed
- Essix and fixed

1. **Factors influencing the choice of a retention protocol:**

- Knowledge gained from orthodontic books
- Clinical experience
- Knowledge and skills gained in postgraduate studies
- Knowledge gained from the Internet (clinical trials and systematic review)
- Other ----------------

1. **Adjudicative treatment you frequently use to increase stability:**

- Over correction
- Interproximal stripping
- Circumferential incision
- None
- Other

1. **Type of retainer mostly preferred by your patients:**

- Essix retainer
- Fixed retainer
- Hawley retainer
- Combination of Essix and fixed retainer
- Combination of Hawley and fixed retainer

1. **Number of checkups after placing a removable retainer?**

- 1
- 2
- 3
- More than 3
- On breakage or damage of the retainer

1. **Initial wearing times of removable retainer:**

- Full time
- After school, evening and night
- Evening and night
- Nighttime only

1. **How long do you usually recommend removable retainer full-time wear?**

- <3 months.
- 3-6 months.
- 7 months - one year.
- >1 year.

1. **Usually when do you stop retention with removable retainers?**

- <2 years after debonding.
- >2 years after debonding.
- after 3rd molars have erupted or extracted.
- wear retainers forever.

1. **Most common indication of fixed retainer is after treatment of:**

- Spacing.
- Crowding.
- Rotation.
- Open bite.

1. **The most preferred fixed retainer used is:**

- Bonded to four incisors.
- Bonded to canines and incisors.
- Bonded from first premolar to first premolar.
- Bonded to canines only.

1. **The most frequent contraindications for fixed retainers:**

- Poor oral hygiene, periodontal problems, caries.
- Occlusion (deep bite).
- Time, cost and maintenance of fixation.
- Patient motivation.
- Other ------

1. **Method of fabrication of the fixed retainer:**

- Directly inside the patient mouth.
- On study cast by orthodontist.
- On study cast by technician.

1. **Usually when do you remove fixed retainers?**

- < 2 years after debonding
- >2 years after debonding
- After 3rd molars have erupted or extracted
- Wear retainers forever

1. **Most common wire used as fixed retainer is:**

- Dead soft multistrand wire
- Multistrand rectangular SS wire
- Multistrand round SS wire
- Chain
- Glass fiber
- Other -------

1. **Number of checkups after placement of fixed retainer:**

- 1
- 2
- 3
- More than 3
- On breakage or debond of the retainer

1. **How often do you usually observe failure of a fixed retainer?**

- Once during the whole retention period
- Twice during the whole retention period
- More than twice during the whole retention period
- Never

1. **Type of bonding material used to fix the fixed retainer?**

- Flowable composite
- Orthodontic bonding material
- Normal composite
